# Zika virus infection induces MiR34c expression in glioblastoma stem cells: new perspectives for brain tumor treatments

**DOI:** 10.1038/s41419-019-1499-z

**Published:** 2019-03-19

**Authors:** Gioacchin Iannolo, Maria Rita Sciuto, Nicola Cuscino, Roberto Pallini, Bruno Douradinha, Lucia Ricci Vitiani, Ruggero De Maria, Pier Giulio Conaldi

**Affiliations:** 1 Department of Research, IRCCS ISMETT (Mediterranean Institute for Transplantation and Advanced Specialized Therapies), Palermo, Italy; 2 Department of Oncology and Molecular Medicine, Istituto Superiore di Sanità, Rome, Italy; 3 Istituto di Neurochirurgia, Università Cattolica del Sacro Cuore, Rome, Italy; 4 Fondazione Policlinico Universitario “A. Gemelli” - IRCCS, Rome, Italy; 5 Regenerative Medicine and Immunology Unit, Ri.MED Foundation at IRCCS ISMETT, Palermo, Italy; 6 Istituto di Patologia Generale, Università Cattolica del Sacro Cuore, Rome, Italy

## Abstract

Zika virus (ZIKV) is a flavivirus with a marked effect on fetal nervous system development. ZIKV treatment has recently been found to also have a benefit against glioblastoma, a highly aggressive brain tumor with a poor prognosis. The reported data do not completely explain the mechanism beyond this effect. Nevertheless, in the majority of the cases no adverse effect has been found in healthy adult humans. In this study, we characterized the ZIKV infection mechanism on glioblastoma stem cells, which are considered responsible for the tumor progression and resistance to conventional therapies. Moreover, we explain why the action of this virus is directed to the stem cells in the nervous system counterpart. Our results confirm the effectiveness of ZIKV treatment against glioblastoma, indicating novel molecular targets that can be introduced for more powerful therapies.

## Introduction

Glioblastoma (World Health Organization grade IV glioma, GBM) is one of the most malignant tumors. It is rapidly fatal, and the current therapies do not have a positive outcome. Glioblastoma patients show, in most cases, a poor response to chemotherapy, followed by tumor recurrence, with a median survival of about 14 months^1^. GBM has a highly undifferentiated phenotype, one of the most important characteristics of this tumor that may account for glioblastoma resistance. In fact, GBM has a high level of stemness, while most of the isolated cells show high multipotency, self-renewal, and an innate protection against apoptosis features. About 15 years ago, a population of cells derived from glioblastoma defined as glioblastoma stem cells (GSCs) was described and characterized^2–4^. These cells represent the tumoral counterpart of the neural stem cells (NSCs)^5^. Several groups have reported a clear resistance in GSCs to radiotherapy and chemotherapy (systematically reviewed by Iannolo et al.^6^). ZIKV is a neurotropic flavivirus that induces fetal microcephaly and infection in pregnant women. The mechanism that induces this effect is poorly understood. Ample evidence indicates that the virus specifically targets the NSC population^7^, causing a massive reduction in neural development. This characteristic has opened the possibility of using it as specific oncolytic virus against GSCs^8^. In this study, we tried to clarify the mechanism responsible for its specific tropism, supporting the indication for its potential use for glioblastoma treatment. Moreover, we found that ZIKV infection in GSCs induces miR34c expression, and that its overexpression reproduces an effect equivalent to the infection.

## Materials and methods

### Cell culture, transfection, and infection

The isolation, culturing, and expansion of GSCs and NSCs has been described previously (GSC#1,#61, #83, #151)^9–12^. GSCs grow as clusters of undifferentiated cells, as indicated by morphology, and express stem cell markers such as CD133, SOX-2, Musashi-1, and nestin. The in vivo tumorigenic potential of GBM neurospheres was assayed by intracranial xenograft in immunocompromised mice.

U87MG and T98G cell lines were obtained by ATCC and cultured, as indicated by the provider. ZIKV, strain H/PF/2013—Asian genotype^13^ was provided by Dr. Giovanni Rezza (Department of Infectious Diseases, ISS Italian National Institute of Health, Rome) under MTA. The virus was propagated using VERO cells (ATCC) in serum-free medium to avoid any induction of GSCs by serum. MiR34c overexpressing vector was obtained with Euroclone (Milan, Italy), small-interfering RNA (siRNA) lentiviral vectors were purchased from Thermo Fisher, (Rockford, IL, USA), and the transduction was done as previously described by Iannolo et al.^14^.

For the growth assays, cells were plated in 6–96 well plates (not treated/low binding, Corning, New York, USA). After the indicated time the cells were collected and lysed with Cell Titer Glo Luminescent 3D Cell Viability Assay reagent (Promega, Mannheim, Germany). Caspase activity was evaluated using the Apotox Triplex Assay (Promega). Luminescence was analyzed using the Spark Microplate Reader (Tekan, Männedorf, Switzerland).

### RNA extraction and reverse transcription PCR (RT-PCR)

Total RNA was purified by miRNAeasy (Qiagen, Germantown, MD, USA) and reverse-transcribed using TaqMan UNIVERSAL MMixII (Applied Biosystems, Waltham, MA, USA) for random priming or MicroRNA (miRNA)-specific assay reverse transcription. Semiquantitative PCR was performed with TaqMan-validated assays (Applied Biosystems): miR34a (000426), hsa-miR-34b (000427), miR34c (000428), hsa-miR-34c-3p (241009_mat). As reference for cDNA, we chose GAPDH (Hs99999905_m1) and U6 (#001973) for miRNA. All analyses were carried out in triplicate. Real-time data were collected using Microsoft Excel, and analyzed with the following formula: Expression level = 2^−ΔΔCt^ method. All experiments were done as independent triplicates and analyzed using standard deviation (SD). The *p*-value was obtained with the Student’s *t*-test.

### NGS analysis

Total RNA was isolated from GSCs infected with ZIKV and compared with uninfected controls. miRNA extraction was done using the miRNeasy Isolation Kit (Qiagen, Hilden, Germany). Sequencing libraries were prepared according to the Illumina Protocol for small RNA (Illumina, San Diego, CA, USA release Feb. 2014). Briefly, one microgram of total RNA was processed using the small RNA library kit, as indicated by the manufacturer (Illumina). The library was loaded in an Illumina MiSeq sequencer in a 51 bp single read mode (Illumina). The data obtained from the sequencer were filtered based on several criteria. As the sequence of the adapter is known, Trimmomatic-0.33^15^ software was used to trim, from the raw data, the adaptors. The sequence reads were then filtered for quality, and clustered in unique sequences to remove redundancy, retaining their individual read count information. Unique sequences 16 nucleotides or more in length were mapped, allowing up to one mismatch on miRNA annotation according to miRBase using Bowtie 0.12.8^16^ software and HTSeq 0.6.0^17^ software for quantification of the expression of each miRNA. This detects the reads corresponding to known miRNAs, giving an estimation of expression level. Identification of differentially expressed miRNAs was made with the Bioconductor DESeq2^18^ package. Starting from the expression values, the first step was to minimize the effect of the systematic technical variations. Then a negative binomial distribution model was used to test differential expression in deep sequencing datasets. Only miRNAs with a *p*-value ≤0.05, and fold-change ≤1.5, or ≥1.5 were considered as differentially expressed. Given the critical roles of miRNAs in regulating gene expression and cellular functions, we predicted their putative targets, intersecting results obtained from mirPath v.3^19^. MirPath provides computationally predicted miRNA gene targets.

### Immunofluorescence

Cell staining was done on GSCs seeded on multichamber slides (Nunclone, Sigma-Aldrich). Cells were washed in PIPES buffer (80 mM PIPES pH 6.8, 5 mM EGTA and 2 mMMgCl2, Sigma-Aldrich, MO, USA), and fixed with 4% paraformaldehyde/PIPES (Sigma-Aldrich) for 10 min. This step was followed by block-permeabilization in PBS containing 0.2% BSA (Sigma-Aldrich) and 0.1% Triton X-100 (Sigma-Aldrich) for 10 min, followed by DAPI staining (Sigma-Aldrich). The antibodies were used at the condition suggested by the suppliers: mouse anti-beta3 tubulin (T8578, 1/100,Sigma)-, rabbit Glial Fibrillary Acidic Protein (GFAP, Z0334, 1/100, Dako, CA, USA), and mouse anti-Flavivirus (Ab10216,1/1000, Abcam, Cambridge, UK).

All microscopic images were acquired with a Nikon system TE2000-S microscope (Nikon Instruments, Amsterdam, Netherlands) equipped with an Olympus LC20 Camera and Olympus soft imaging LCmicro software (Olympus, Milan, Italy), or with a Leica confocal station (Leica SP5 confocal system, mounted on a Leica DM6000 inverted microscope, equipped with an Argon-ion laser and PMT detectors) (Leica Microsystems, Wetzlar, Germany).

### Immunoblotting

Cells were lysed with a buffer containing 1% Triton X-l00, 50 mM HEPES (pH 7.5), 150 mM NaC1, 10% glycerol, 1.5 mM MgCl2, 5 mM EGTA, protease inhibitors (4 mM phenyl methylsulfonyl fluoride and 100 mg/ml aprotinin, Sigma-Aldrich), phosphatase inhibitors (10 mM sodium orthovanadate and 20 mM sodium pyrophosphate, Sigma-Aldrich) and processed. For direct immunoblot analysis, we employed 15–30 μg of total cellular proteins, re-suspended with 25 μl of loading buffer, boiled for 5 min and loaded on sodium dodecyl sulfate polyacrylamide gel electrophoresis (SDS-PAGE) for western blot (WB). The antibodies for WB were used at the condition suggested by the suppliers: rabbit anti-NOTCH-1 (Ab27526, 1/500, Santa Cruz Biotechnology, Texas, USA), rabbit anti-p73 (Ab189896, 1/1000, Abcam), rabbit anti-Bcl2 (Ab185002, 1/500, Abcam), rabbit anti-human NUMB (ab-14140, 1/1000, Abcam), goat anti-AKT (C-20) (1/500, sc-1618, Santa Cruz), rabbit anti-Phospho-Akt-Ser-473 (#9271 1/500, Cell Signaling), and mouse anti-beta-actin (sc-81178, 1/1000, Santa Cruz Biotechnology).

### Statistical analysis

Expression values of numb mRNA in normal tissues and in glioma samples, distributed by histology classification, with a cutoff equal to the median (*p*-value with Bonferroni) were downloaded from GlioVis Data Portal^20^. The Kaplain–Meier survival data, high NUMB vs. low expressing samples in all subsets or only in GBM samples were considered. The median cutoff and the z-score were provided by Gliovis, considering all subsets or only glioblastoma.

## Results

**Fig. 1:**
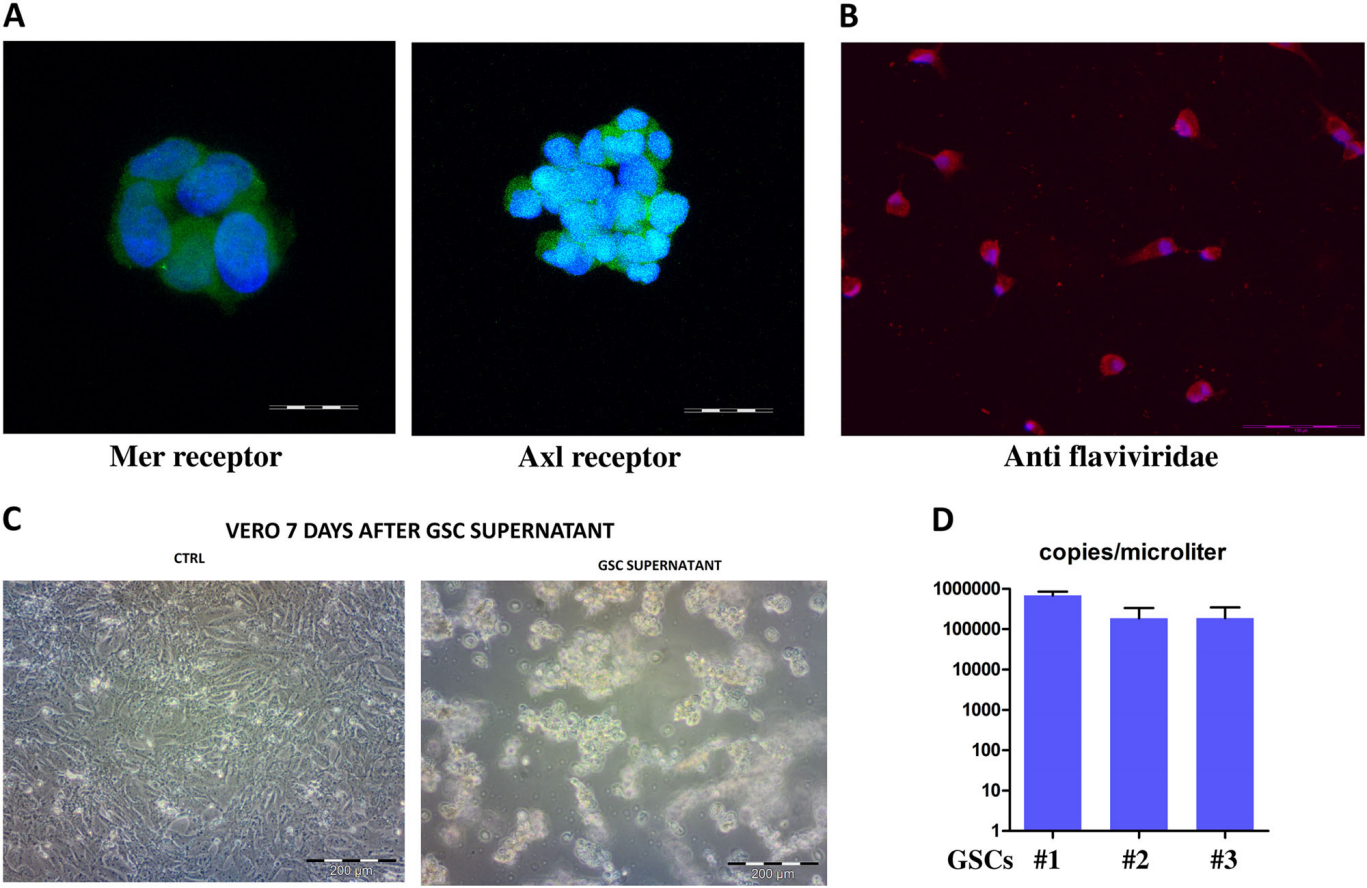
Evaluation of ZIKV’s ability to enter and replicate in GSCs. **a** Immunofluorescence of Glioblastoma Stem Cells (GSCs; spheres) stained with Mer Receptor (left, green), Axl Receptor (right, green), and DAPI for nuclei staining (blue)(scale bar 20  μm). **b** GSC immunofluorescence (single cells) after Zika infection stained with an anti-flaviviridae antibody (red) and DAPI for nuclei (blue). **c** Vero cells infected with GSC-derived supernatant (right) vs. control (left). **d** Quantitative analysis of the ZIKV supernatant derived from GSCs

ZIKV (ZIKV-strain Asian genotype-) was used to establish its action on human GSCs. The cells used have been previously tested for their ability to form tumors and for the presence of stemness markers^9–12^. We verified the presence of the putative Mer and AXl Zika receptors by immunofluorescence (IF) analysis, in which it was possible to assess that GSCs express both receptors (Fig. 1a).

**Fig. 2:**
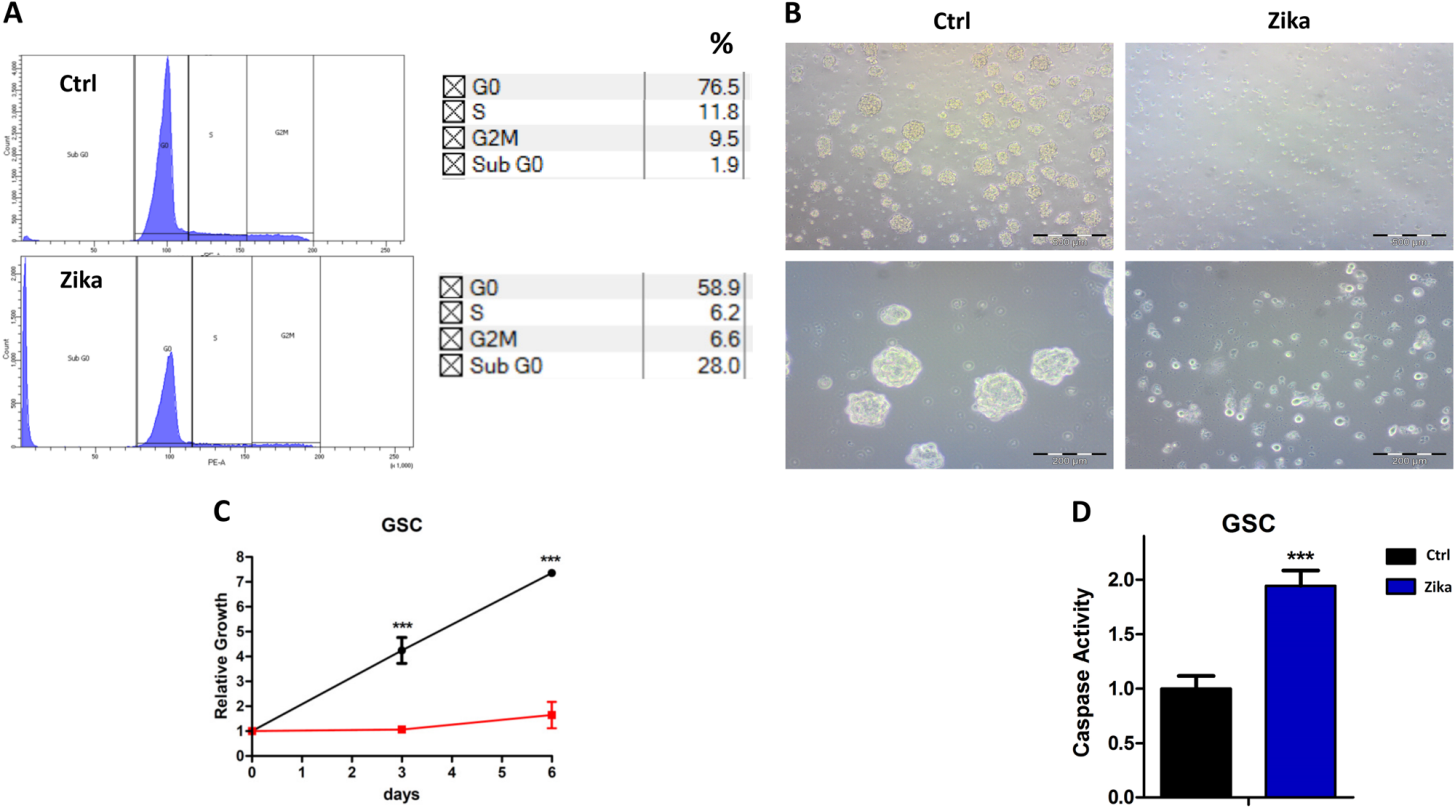
Apoptotic effect of Zika infection in GSCs. **a** Propidium Iodide (PI) FACS analysis of Zika infection in glioblastoma stem cells (GSCs; one representative experiment is shown). **b** Morphological analysis of GSCs after 3 days of Zika infection (bright field, two magnifications) (**c**). Growth rate of GSCs after Zika infection evaluated by Cell Titer Glo. **d** Caspase activity evaluated by luminescence after ZKV infection (all reported experiments were carried out in triplicate on three different GSC clones)

Then, we evaluated the ZIKV’s ability to enter and replicate in these cells by IF analysis. Using an anti-Zika antibody, we were able to observe a bright fluorescence in all the infected cells (Fig. 1b). To demonstrate the replication activity and the production of infective viral particles, we tested the GSC-derived supernatant by infecting the VERO cell line (Fig. 1c), and the titrated virus particles by RT-PCR quantitative TaqMan assay (Fig. 1d). The virus was clearly present in the supernatant and was able to infect the target cells. To further evaluate the effect of the Zika infection on GSCs, we carried out a propidium iodide (PI) cell cycle analysis by flow cytometry, and found an increase of apoptosis as measured by the sub G0 shift (Fig. 2a). The ZIKV infection reduced the formation of spheres, as shown by microscopy analysis (Fig. 2b), and it was possible to assess a clear cell growth reduction by Cell Titer Glo (Promega) (Fig. 2c). Moreover, we observed an enhancement of caspase activity due to the infection (Fig. 2d).

**Fig. 3:**
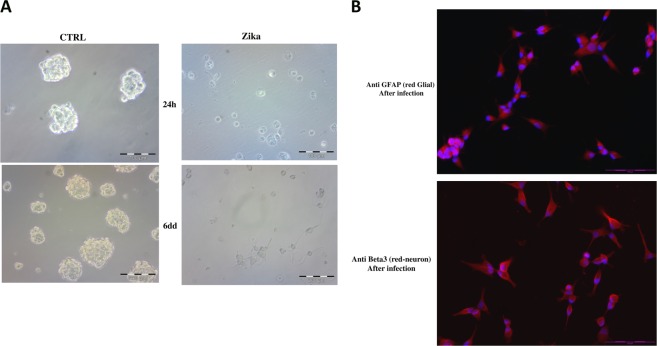
Analysis of the GSCs differentiation induced by Zika infection. **a** Morphological analysis of non-treated and Zika-infected glioblastoma stem cells ((GSCs); untreated plates, bright field) after 1 day and 6 days. **b** Immunoflurescence analysis after Zika infection. The treated cells were induced to adhere (non-treated chambers). In Red GFAP (glial staining, upper panel) and Beta III Tubulin (neuronal staining, lower panel) and in blue DAPI (nuclei)

**Fig. 4:**
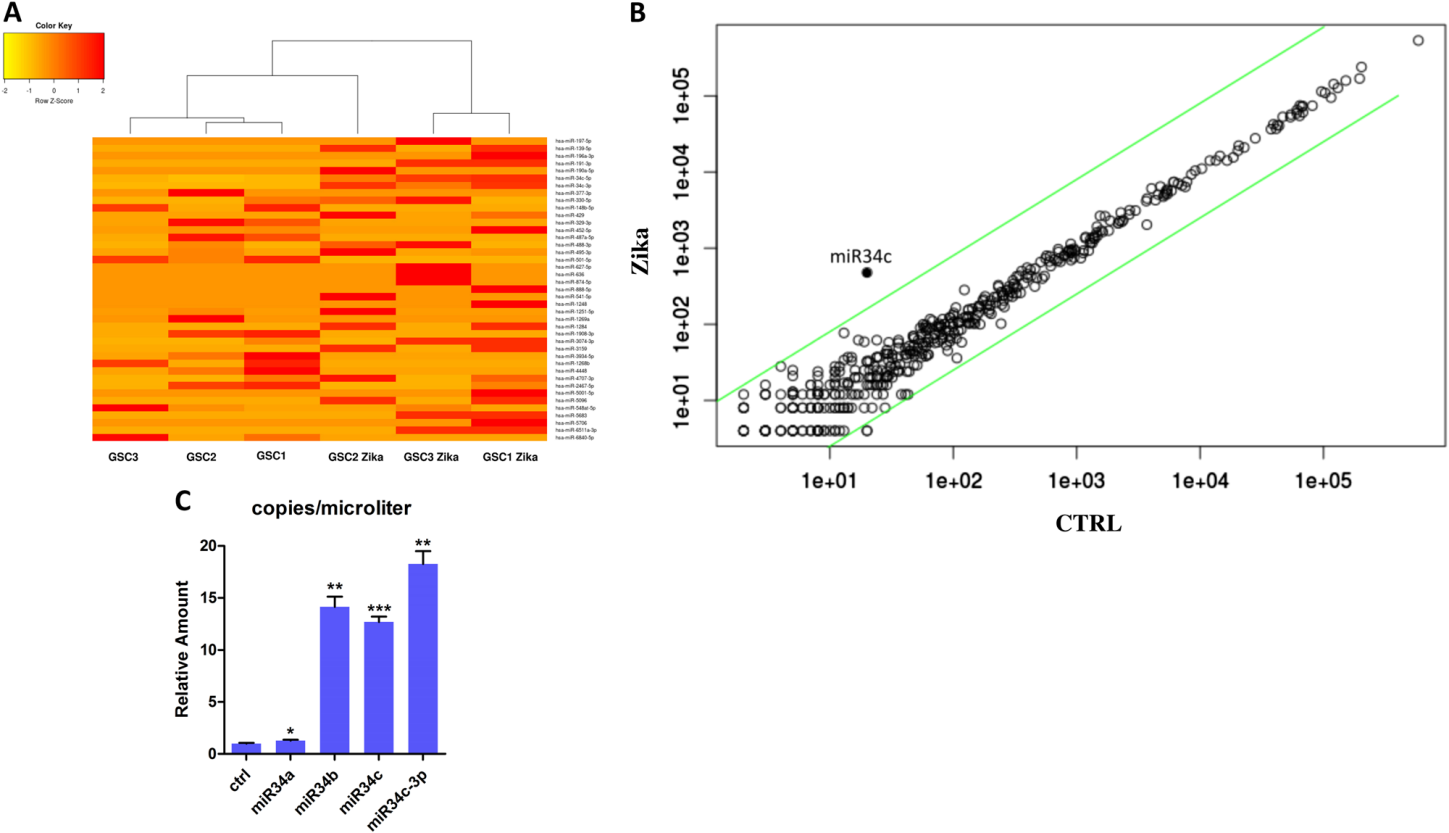
MiRNAs expression analysis of Zika-infected GSCs. **a** Heat map representing miRNA clusterization from NGS data. Expression data have been scaled to have a mean zero and standard deviation one fragment per million, on the row average value (in yellow lower, in red higher values). **b** Scatter plot analysis on the *x*-axis are the mean expression for controls, and the *y*-axis the miRNAs expressed by Zika-infected cells. **c** TaqMan assay for miR34 family expression (all reported experiments were carried out in triplicate on three different GSC clones)

It is important to note that disaggregated GSC cells showed an increase in substrate adhesion, also in the non-treated plastic, assuming a more differentiated shape after ZKV infection (Fig. 3a). The adherent cells were evaluated by IF for differentiation markers (Fig. 3b), confirming that the GSCs displayed the same behavior as the non-stem counterpart after infection, with an induction to differentiate^21^. To dissect the Zika viral infection cascade, we performed a microRNA analysis by next-generation sequencing (NGS), which identified a clear clusterization between the treated and untreated samples (Fig. 4a). The analysis of the miRNA expression in Zika-infected GSCs vs. control cells showed (Fig. 4b) that miR34c is one of the most promising upregulated miRNA. To confirm the differences in the miR34c expression induced by Zika infection, we did a real-time PCR (Taqman assay) on three different clones. We found an enhancement of miR34c expression in all Zika-infected cells, confirming the NGS results (Fig. 4c).

**Fig. 5:**
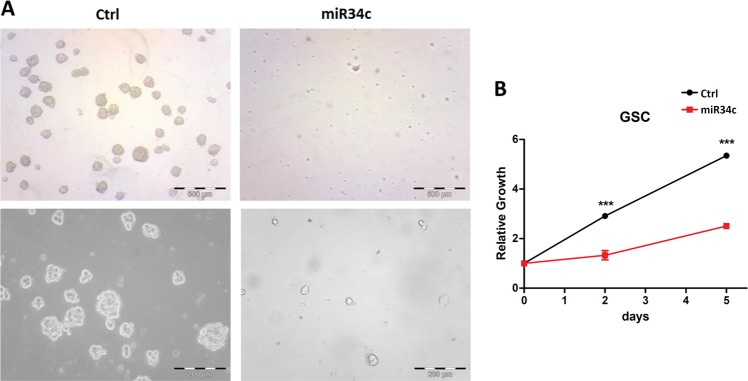
MirR-34c overexpression in GSCs. **a** Morphological analysis of glioblastoma stem cells (GSCs) overexpressing miR34c vs. control (bright field, two magnifications). **b** GSC growth curve after miR34 overexpression vs. control evaluated by Cell Titer Glo (all reported experiments were carried out in triplicate on three different GSC clones)

**Fig. 6:**
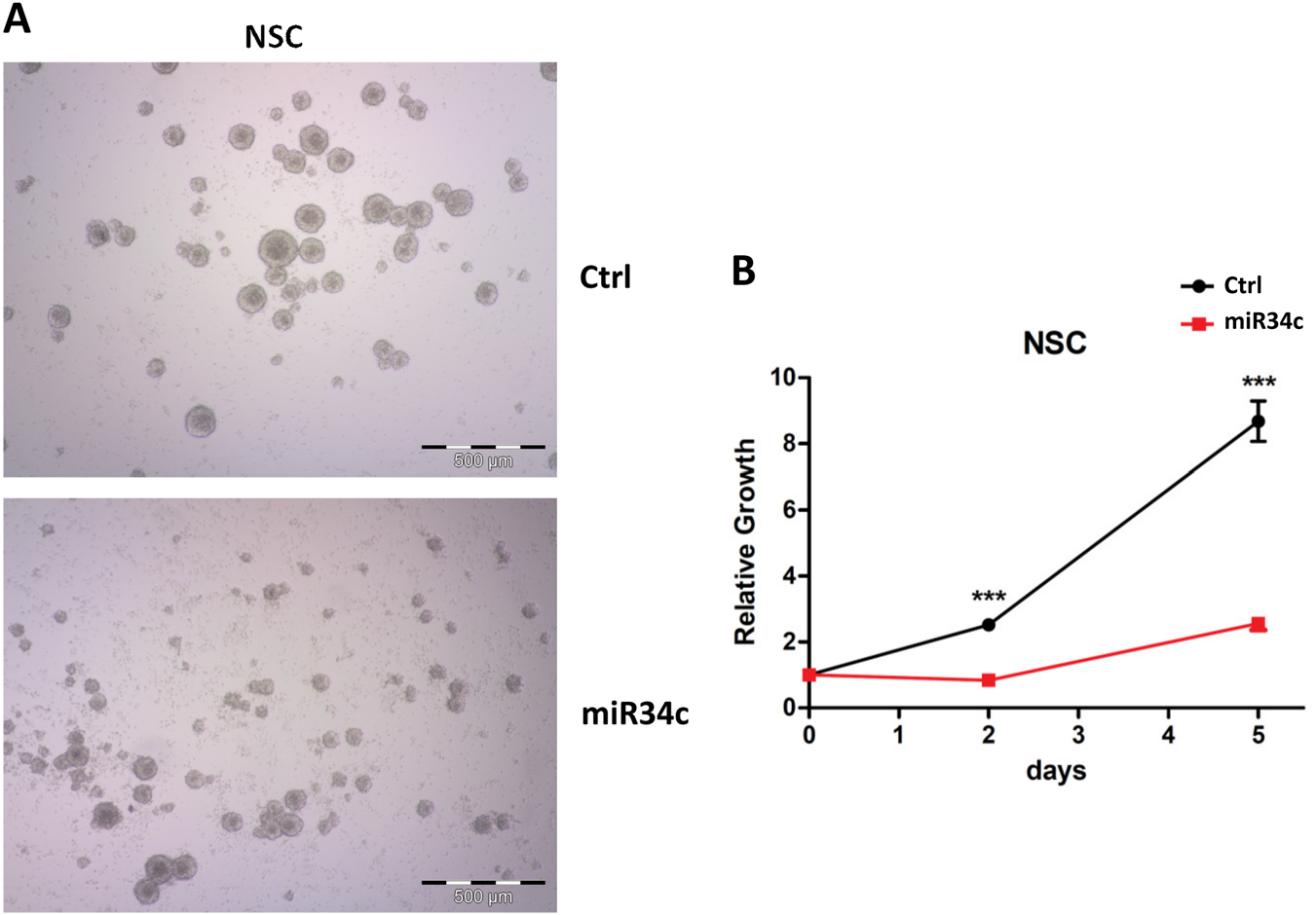
Effect of miR-34c overexpression in NSCs. **a** Morphological analysis of neural stem cells (NSCs) overexpressing miR34c vs. control (bright field). **b** NSC growth curve after miR34 overexpression vs. control evaluated by Cell Titer Glo (all reported experiments were carried out in triplicate)

To evaluate the miR34c upregulation effect found after ZIKV infection, we overexpressed this miRNA in GSCs. The overexpression reduced the sphere formation (Fig. 5a) and, as in the Zika infection, in non-treated plates the single cells are induced to adhere, presenting a more differentiated shape (Fig. 5a). The growth reduction after miR34c overexpression was confirmed by Cell Titer Glo (Fig. 5b). Pairwise, we observed the same response after miR34c overexpression in the normal counterpart (NSCs), with sphere formation (Fig. 6a) and cell growth reductions (Fig. 6b).

**Fig. 7:**
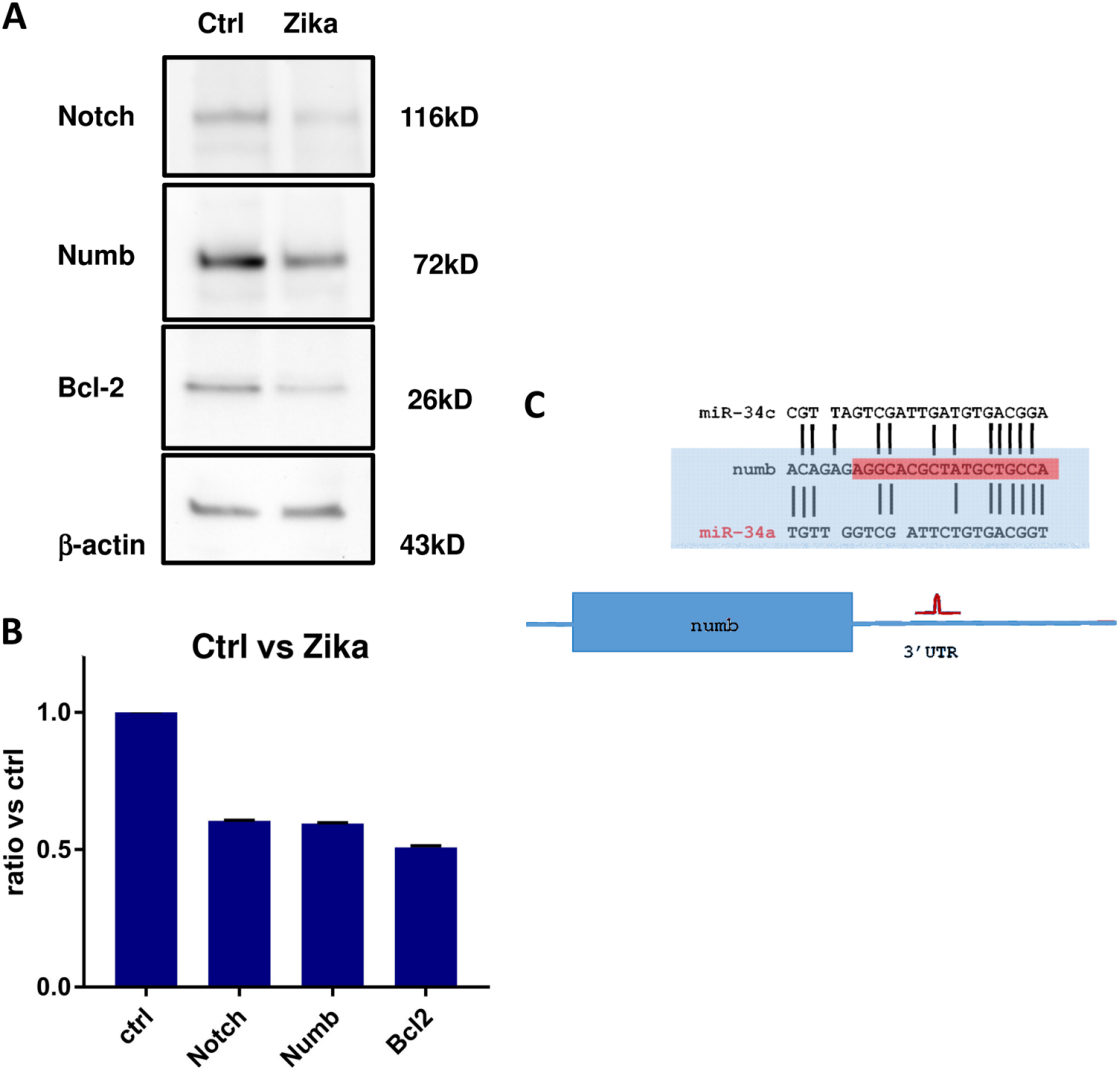
Analysis of protein expression after Zika infection. **a** Western blot (WB) analysis for the indicated proteins on glioblastoma stem cells (GSCs) infected with Zika vs. control and **b** protein quantification. **c** Comparison of the miR34a and mir34c regions (reported by previous authors^25^ binding the 3′ untranslated of Numb mRNA. It is possible to observe an analogy between the binding sites between the two miRNAs

**Fig. 8:**
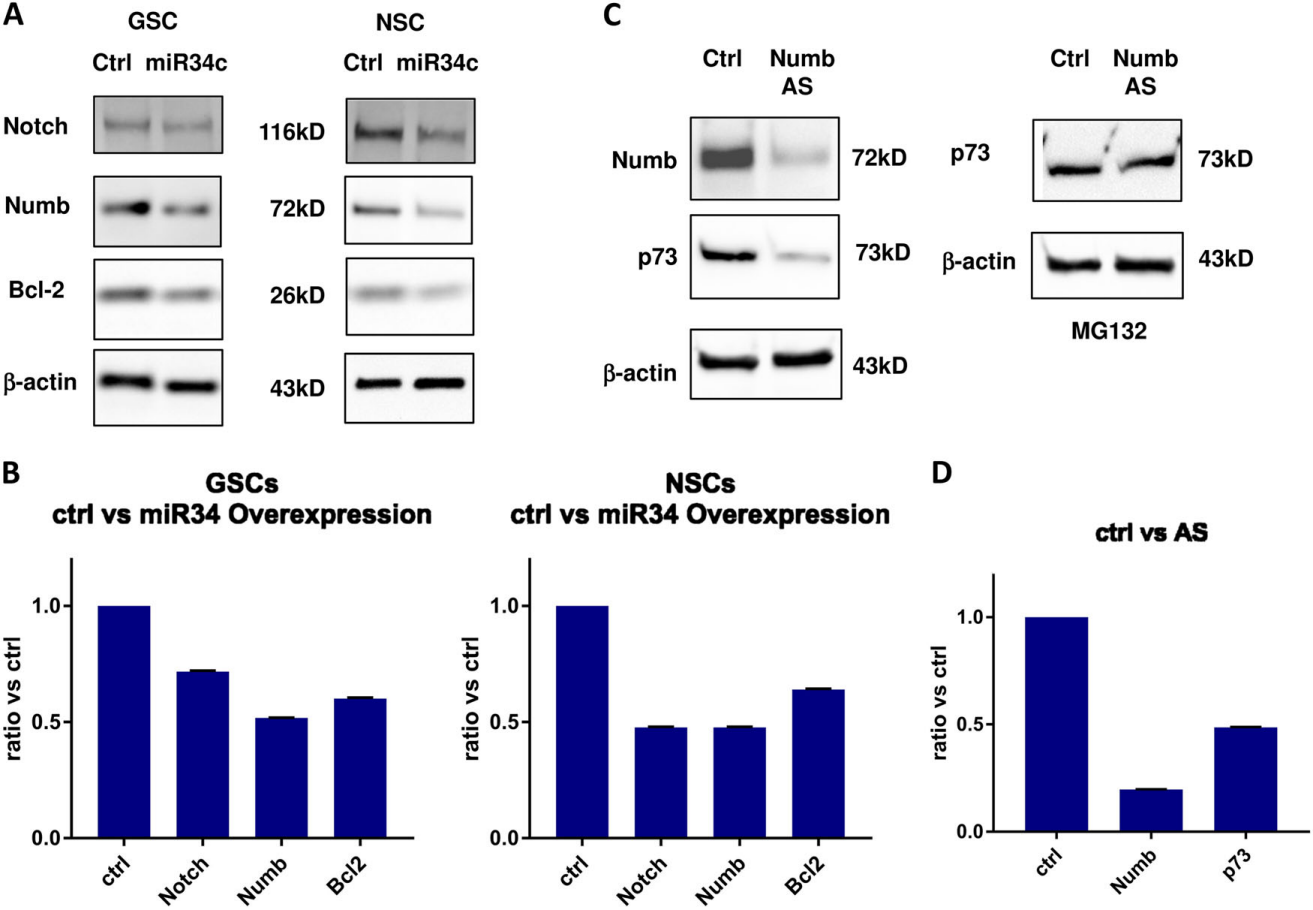
MiR34c influence on protein expression. **a** Western blot (WB) analysis of the indicated proteins on neural stem cells (NSCs) and glioblastoma stem cells (GSCs) overexpressing miR34c vs. control and **b** protein quantification. **c** (left) Analysis of the indicated proteins on T98G down-modulated for NUMB (**d**) and protein quantification. **c** (right) Western blot analysis of p73 expression reverted by MG132 treatment

Mir34 acts on the regulation of different genes, such as Bcl2, involved in the inhibition of the apoptotic pathway^22^, and genes such as NOTCH and NUMB, involved in stemness maintenance and in nervous system development^22–24^. For this reason, we evaluated the expression of these proteins by Western blot analysis after ZIKV infection. As expected, there was a reduction in all the considered proteins (Fig. 7a, b). The effect of miR34c on NUMB expression can be explained through the interaction on the 3′ untranslated region of NUMB mRNA marked in light blue^25^ (Fig. 7c). It was possible to assess that the region was conserved among miR34a and miR34c. To confirm the miR34c action in these cells, we used a lentiviral overexpressing vector and evaluated the effect on Bcl2 and NUMB protein expression. For both proteins there was a significant reduction after miR34c overexpression (Fig. 8a, b), as found in both GSCs and normal NSCs reflecting the effect of ZIKV infection (Fig. 7a).

To dissect the possible cascade triggered by mi34c overexpression, we evaluated other proteins associated with NUMB down-modulation. In our previous study^26^, we observed that NUMB expression reduces p63 proteosomal degradation. This action is mediated through Itch binding. As p63 protein, p73 is degradated through the proteasome by Itch interaction. P73 belongs to the p53 family and its expression in glioblastoma confers invasiveness and chemoresistance. We tested whether in glioblastoma cells lines NUMB down-modulation induces p73 proteosomal degradation. We then down-modulated NUMB by antisense in T98G cell line and evaluated whether p73 expression is NUMB-dependent. As evident from the protein analysis, p73 expression directly correlated with NUMB expression (Fig. 8c, left, 8d). Moreover, p73 down-modulation induced by antisense treatment was reversed by the MG132 proteasome inhibitor (Fig. 8c, right). This clearly defined a proteosomal-dependent mechanism, as described in a previous study on p63 in the epithelium^26^.

**Fig. 9:**
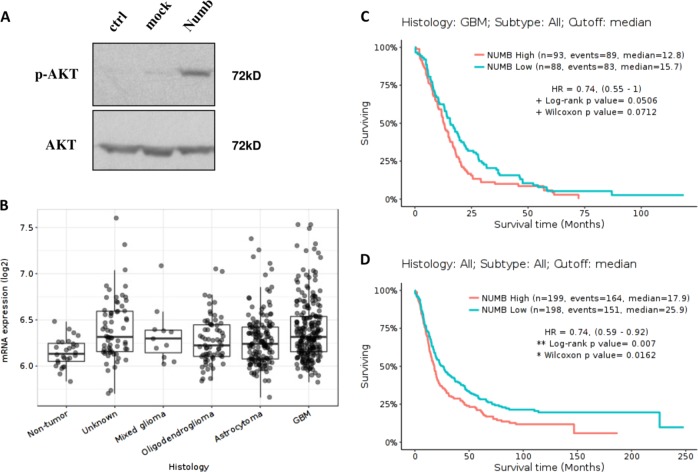
Numb impact in glioblastoma. **a** Western blot analysis for the indicated proteins on U87MG cells overexpressing NUMB. **b** NUMB expression analysis in normal and glioma samples (mixed glioma, oligodendroglioma, astrocytoma, and GBM) of the TCGA data (*p* < 0.001). **c** Survival analysis of NUMB high vs. low expressers within the GBM subtype of TCGA or all subtypes (**d**). Patient data were downloaded from the GlioVis data portal. High and low were defined as cutoff equal to the median, and all Kaplan–Meier plots were analyzed using Log-rank statistics and Wilcox *p-*values

To better dissect the NUMB function in glioblastoma, we overexpressed it in the U87MG cell line, negative for PTEN^27^. In this cellular system, NUMB overexpression induced AKT phosphorylation in Ser-473. This phosphorylation site has been shown to be a key activator of AKT^28^, suggesting a possible involvement of NUMB in tumor progression (Fig. 9a). To evaluate this possibility we analyzed NUMB expression in normal and glioma tumors and correlated its expression with patients’ survival data using the GlioVis portal database. This evidenced an inverse prognosis between NUMB expression and glioblastoma (Fig. 9b–d), indirectly confirming the role of NUMB in glioblastoma cell survival.

## Discussion

This study clearly confirms the effect of ZIKV on human GSCs. Viral infection induces an increase in cell death and a striking apoptotic response assessed by sub G0 induction, with an increase of caspase activation. Moreover, for the first time, to the best of our knowledge, we have elucidated the putative mechanism that causes the Zika effect on NSCs and GSCs. This effect can be ascribed to the intrinsic nature of these cells, which need the maintenance of specific developmental pathways for their growth. The virus interferes with the stem cell maintenance/proliferation by inhibiting developmental-related genes, in particular NOTCH and NUMB. Both of these proteins have been widely involved in normal central nervous system development, and can offer an explanation for the Zika effect on normal brain development^22–24^. Moreover, we observed that Zika infection reduces Bcl2 expression, which can be responsible for the apoptotic response, together with the reduction of NUMB, decreasing AKT phosphorylation in glioblastoma cell lines. We also found that NUMB down-modulation induces p73 degradation in a proteosomal-dependent manner. P73 was found to confer an invasive phenotype in glioblastoma cells, and its deletion impairs the invasiveness and the chemoresistance in both animal models and glioblastoma patients, with a prolonged survival (REMBRANT database NIH: https://caintegrator.nci.nih.gov/rembrandt/)^29^. Pairwise, we observed that NUMB expression inversely correlates with glioblastoma patients’ survival.

The NGS analysis evidenced that miR34c expression is clearly induced by ZIKV infection in GSCs. We found that ZIKV could exert these effects by inducing the expression of miR34c, which regulates all these proteins. Moreover, the expression of miR34c reduces NSCs and GSCs cell growth and regulates both Bcl2 and NUMB expression, mimicking the same effect observed in the ZIKV infection. The MiR34 family has been correlated at various levels with senescence, differentiation, and apoptosis by regulating different genes. We found that these genes are regulated in NSCs and GSCs by miR34 overexpression, reducing cell growth. MiR34 clearly inhibits Bcl2, which is involved in the resistance to the apoptosis in enhanced cell survival and in response to radiation (systematically reviewed by Iannolo et al.^6^). Apoptotic resistance can also be influenced by NUMB-inducing AKT phosphorylation. In particular, we found that miR34c, like ZIKV infection, reduces NUMB expression in both NSCs and GSCs.

NUMB expression was also correlated with poor prognosis, and in particular, its down-modulation is also involved in p73 proteosomal degradation, where p73 is involved in both the maintenance of neural stem/progenitor cell self-renewal and differentiation regulating SOX-2, Hey-2, TRIM32 and NOTCH transcription (systematically reviewed by Victoria Niklison-Chirouet et al.^30^). The results of our study confirm that ZIKV could potentially be used as an oncolytic virus in glioblastoma treatment, while it remains to be studied whether ZIKV can elicit adverse effects in glioblastoma patients. ZIKV infection is normally asymptomatic. However, in rare cases, it can cause adverse reactions, such as fever, rash, myalgia, headache and conjunctivitis. More important, but still limited, are various neurologic complications in adults such as Guillain-Barré syndrome, meningoencephalitis, myelitis, and ophthalmologic abnormalities^31^. However, it must be considered that the use of ZIKV can be more aggressive when the brain barrier is compromised.

By dissecting ZIKV action on GSCs, we provide an alternative tool for glioblastoma treatment, and demonstrate that mir34c can inhibit GSCs, such as ZIKV, by reducing Bcl2 that could potentially increase the effect of chemo/radiotherapies. Mir34 overexpression has been used in both multiple myeloma^32^, melanoma^33^, and colon cancer^20^. Future studies should test whether miR34c inhibition could be successfully employed in ZIKV-infected patients to reduce neural defects in fetal development.
